# Bovine Spongiform Encephalopathy Infectivity in Greater Kudu (*Tragelaphus strepsiceros*)

**DOI:** 10.3201/eid1006.030615

**Published:** 2004-06

**Authors:** Andrew A. Cunningham, James K. Kirkwood, Michael Dawson, Yvonne I. Spencer, Robert B. Green, Gerald A.H. Wells

**Affiliations:** * Institute of Zoology, Regent’s Park, London, United Kingdom;; † Veterinary Laboratories Agency, Addlestone, Surrey, United Kingdom

**Keywords:** Bovine Spongiform Encephalopathy, BSE, greater kudu, *Tragelaphus strepsiceros*, bovine, tissue distribution, transmission

## Abstract

Of all the species exposed naturally to the bovine spongiform encephalopathy (BSE) agent, the greater kudu (*Tragelaphus strepsiceros*), a nondomesticated bovine from Africa, appears to be the most susceptible to the disease. We present the results of mouse bioassay studies to show that, contrary to findings in cattle with BSE in which the tissue distribution of infectivity is the most limited recorded for any of the transmissible spongiform encephalopathies (TSE), infectivity in greater kudu with BSE is distributed in as wide a range of tissues as occurs in any TSE. BSE agent was also detected in skin, conjunctiva, and salivary gland, tissues in which infectivity has not previously been reported in any naturally occurring TSE. The distribution of infectivity in greater kudu with BSE suggests possible routes for transmission of the disease and highlights the need for further research into the distribution of TSE infectious agents in other host species.

To date, 13 species of zoo animal have been confirmed as having died with a novel scrapie-like spongiform encephalopathy (SE) concurrent with the bovine spongiform encephalopathy (BSE) epidemic (Table 1). The disease is thought, in some, if not all, of these species, to be caused by infection with the BSE agent. In addition, natural infection with BSE has been reported in five species of primate in French zoos (*11*), but these results are considered equivocal for the confirmation of a spongiform encephalopathy (12, G.A.H. Wells, unpub. data). BSE was diagnosed in six of eight greater kudu (*Tragelaphus strepsiceros*), a member of the family Bovidae, subfamily Bovinae, that died at the London Zoo from 1989 through 1992 (*2**,**13**,**14*). The epidemiology of this disease in greater kudu is consistent with either a particularly high susceptibility to infection, the occurrence of direct animal-to-animal transmission of the disease, or with a combination of these factors (*2**,**14**,**15*). To investigate further the biology of BSE in greater kudu, the distribution of the infectious agent in greater kudu with BSE was determined by using the mouse bioassay method.

**Table 1:** Species of zoo animal with confirmed novel spongiform encephalopathy required contemporaneously with epidemic of bovine spongiform encephalopathy in domestic cattle^a^

Species	No. of cases	Reference
*Bovidae*		
Nyala, *Tragelaphus angasi*	1	1
Greater kudu, *Tragelaphus strepsiceros*	6	2
Gemsbok, *Oryx gazella*	1	1
Arabian oryx, *Oryx leucoryx*	1	3
Scimitar-horned oryx, *Oryx dammah*	1	2
Eland, *Taurotragus oryx*	6	2,4
American bison, *Bison bison*	1	5
*Felidae*		
Cheetah, *Acinonyx jubatus*	10^b^	2,5–8
Puma, *Felis concolor*	3	2,5
Ocelot, *Felis pardalis*	3	4,8
Tiger, *Panthera tigris*	3	5
Lion, *Panthera leo*	4	4,5
Asian golden cat, *Catopuma temminckii*	1^c^	10

^a^Animals were born and cases occurred in Great Britain unless stated otherwise. ^b^The initial case of transmissible spongiform encephalopathy in cheetah occurred in Australia, one case in the Republic of Ireland, and three cases in France; all animals were born in Britain except the most recently reported case in France (*8*). ^c^Reported from Australia, born in Germany, and kept for a period in the Netherlands (*10*).

## Materials and Methods

### Tissues

Tissues from four greater kudu that died with spongiform encephalopathy were tested for infectivity by bioassay in C57Bl-J6 mice (Table 2). The epidemiologic, clinical, and pathologic findings of the disease in the kudu have been described in detail previously (*2**,**14**–**16*), and a summary of the relevant details is given in Table 3. Tissues for bioassay were collected principally from kudu A1212; each sample was collected in a sterile container by using new disposable instruments and gloves to prevent cross contamination between tissues. As TSE infectivity has been demonstrated previously by bioassay in tissues preserved in formalin and paraffin wax (*13**,**17**,**18*), tissue samples obtained opportunistically after routine postmortem examinations of three additional kudu (A664, A666, and A1221) were also tested for infectivity by using mouse bioassay. Samples collected from kudu A666, A1212, and A1221 were stored in separate, sterile containers and were either frozen at –70°C or fixed in neutral buffered 10% formalin. Non-neural tissues from kudu A664 were fixed in neutral buffered 10% formalin in a common container. The brain from this animal was the last organ removed at necropsy and was fixed in 10% formol saline in a separate container.

**Table 2:** Bioassay results for greater kudu tissues injected into C57Bl-J6 mice

Kudu identification no.	Tissue^a^ injected	Positive mice/total^b^	Mean survival period post injection (days) ± SD^c^	Survival period range (d)^c^
A1212	Rostral cerebrum	13/20	595±84	428–745
	Cranial thoracic spinal cord	19/20^d^	557±121	413–821
	Lumbar spinal cord	15/19	521±69	432–634
	Spleen	3/11^d^	819±41	773–851
	Retropharyngeal lymph node	6/11^d^	784±77	691–921
	Popliteal lymph node	0/20^d^	N/A^e^	N/A
	Visceral lymph nodes (pool)	14/18	622±114	448–860
	Submandibular lymph node	0/20^d^	N/A	N/A
	Distal ileum	11/20	547±98	426–718
	Lung	1/14^d^	N/A	746
	Kidney	0/18	N/A	N/A
	Caruncular endometrium	0/20^d^	N/A	N/A
	Ovary	0/20	N/A	N/A
	Mammary gland	0/20^d^	N/A	N/A
	Submandibular salivary gland	1/17^d^	N/A	599
	Conjunctiva	1/16^d^	N/A	659
	Nasal mucosa	0/20	N/A	N/A
	Skeletal muscle (biceps brachii + vastus lateralis)	0/19^d^	N/A	N/A
	Skin (flank)	2/18^d^	713±22	697–728
	Feces	0/11	N/A	N/A
	Serum	0/19	N/A	N/A
A664	Spleen (P)	1/8^d^	N/A	929
	Visceral lymph nodes (pool) (P)	6/15^d^	843±113	649–952
	Lung (P)	0/20	N/A	N/A
	Kidney (P)	0/20	N/A	N/A
	Skeletal muscle (P)	0/19	N/A	N/A
A1221	Brainstem	10/18	634±62	541–762
	Kidney	0/20	N/A	N/A
	Skeletal muscle (vastus lateralis) (F)	0/19	N/A	N/A
A1221 + A666 (pool)	Spleen (pool)	0/20	N/A	N/A
	Visceral lymph nodes (pool)	10/18	700±108	455–851
	Lung (pool) (F)	0/19	N/A	N/A

^a^Tissues not prepared fresh are suffixed: (F), fixed, (P), paraffin wax embedded. ^b^Number of mice positive/number of mice surviving when the first mouse was confirmed positive by histopathologic or immunochemical examination. The denominator for negative groups is the number of mice examined. ^c^Survival periods of positive mice determined positive either by histopathologic or immunohistochemical examination results. ^d^Mice examined by PrP^Sc^ immunohistochemical examinations. ^e^N/A, not applicable.

**Table 3:** Details of spongiform encephalopathy–positive greater kudu used for mouse inoculation studies

Kudu ref. no.	Age at death (mo)	Sex	Brief history	Basis of diagnosis
A664	30	F	Born at London Zoo. Died after progressive neurologic disease of approximately 72 hours. Examined postmortem on the same day.	Histopathologic examination of brain and experimental transmission to mice
A666	37	M	Born at London Zoo. Killed after progressive neurologic disease of approximately 24 hours. Examined postmortem on the same day.	Histopathologic, SAF, and PrP^Sc^ immunocytochemical examinations of the brain and spinal cord^a^
A1221	18	M	Born at London Zoo. Killed for management reasons as a clinically healthy animal and immediately examined postmortem.	Histopathologic, SAF, and PrP^Sc^ immunocytochemical examinations of the brain and spinal cord
A1212	39	F	Born in Britain, moved to London Zoo at 12 months of age. Killed following progressive neurologic disease lasting approximately 1 month and immediately examined postmortem.	Histopathologic and SAF examinations of the brain

^a^SAF, scrapie-associated fibrils; PrP^Sc^, transmissible spongiform encephalopathy disease–specific form of the PrP protein.

Previously, infectivity had been detected in formalin-fixed brain tissue from kudu A664 by bioassay using five inbred mouse strains, with similar incubation periods and lesion profiles to those demonstrated for the BSE agent from domestic cattle with BSE (*13*). Bioassay of brain from kudu A664 was not repeated in the current study, but a positive control sample of fresh brain tissue from clinically affected kudu A1212 was tested.

### Bioassay

The tissues injected into mice for BSE-bioassay are listed in Table 2. Most of the tissue homogenates were prepared from thawed samples of fresh tissues frozen at –20°C. Tissue homogenates prepared from formalin-fixed tissues were rinsed overnight in running water to leach out the fixative, while formalin-fixed, paraffin-embedded tissues were dewaxed in chloroform (two changes) and washed in several changes of absolute alcohol before being rehydrated by immersion in a series of aqueous solutions of descending concentrations of alcohol, through to 100% water. Material for each tissue homogenate was dissected from the center of each tissue sample by using single-use disposable instruments and rigorous sterile procedures. Each sample was homogenized in 10% physiologic saline to make a 10% wt/vol suspension, which was then passed through a gauze filter. To tissue homogenates containing distal ileum or feces, ampicillin was added at the rate of 1.25 mg/mL.

For each tissue homogenate, 20 C57Bl-J6 mice (4–7 weeks old) were each injected by the intracranial route (0.02 mL) and by the intraperitoneal route (0.10 mL). Single tissue or pooled tissue samples were prepared and injected into C57Bl-J6 mice for a standard qualitative assay of infectivity (*13**,**19*).

Mice injected with different tissue or tissue-pool homogenates were housed in separate cages. Injected mice were coded, and detailed clinical monitoring of the mice was carried out by using a standard protocol. The clinical endpoint was determined when mice either showed clear signs of neurologic disease (*20*) or other deterioration of health. Surviving mice were killed 950 days after injection. Postmortem confirmation of disease in mice was routinely carried out by histopathologic examination of the brain for morphologic changes of spongiform encephalopathy.

After the histopathologic assessment of mice, immunohistochemical examination (IHC) for evidence of spongiform encephalopathy disease-specific PrP (PrP^Sc^) was performed on the brains of all mice in selected tissue groups. The groups were mice in which either a low number were positive, testing was inconclusive on histopathologic assessment, or the results indicated a novel or anomalous distribution of the agent in kudu compared to that in other TSE. Additional groups of interest (skeletal muscle, endometrium, and mammary gland), which were negative on the histopathologic examination of mouse brains, were also examined by IHC. Immunohistochemical detection of PrP^Sc^ was introduced to the standard protocol to improve specificity and sensitivity of detecting BSE transmission to mice (*21**,**22*) and interpret inconclusive histopathologic results (*23*). For control purposes, the brains from mice that had been injected with pathologically affected cranial thoracic spinal cord from kudu A1212 were also immunostained. Brains from normal mice that were not injected with infected tissues were similarly examined to provide negative controls.

The immunohistochemical method used was essentially that applied previously to cattle central nervous system tissues (*24*) and adapted for use in mouse brain tissue. Anti-bovine PrP^Sc^ serum (971) was used at 1/8,000 and 1/16,000 dilutions in an avidin-biotin-peroxidase (ABC) complex technique. Transmission was defined by histopathologic evidence of spongiform encephalopathy, or, where applied, immunohistochemical presence of disease-specific PrP (PrP^Sc^) in the brains of the mice.

## Results

The results of the bioassay of tissues are given in Table 2. Based on the histopathologic examination of mouse brains, 15 of the 32 tissue homogenates were positive. The nine histopathologically positive groups examined immunohistochemically were confirmed positive by this method with marginal improved sensitivity of detection (2–3 more mice positive) in only three groups. For nine of the positive tissue homogenates, prepared from fresh central nervous, lymphoreticular, or distal ileum tissue, the proportion of positive mice (>40%) indicated moderate or high levels of spongiform encephalopathy infectivity. The remaining six positive groups (Table 2) had low proportions of positive mice (6%–27%), indicative of relatively low titres or only traces of infectivity. Low numbers (1–2) of histopathologically inconclusive mice in five tissue homogenate groups, which included two groups (A1212, popliteal and submandibular lymph nodes) that contained no histopathologically positive mice, were resolved almost exclusively as negative when examined using immunohistochemistry. The one exception was an inconclusive mouse in A1212 retropharyngeal lymph node group, which proved immunohistochemically positive.

## Discussion

Fifteen of the 32 kudu tissue homogenates transmitted BSE to mice. The positive result for brain tissue from kudu A1221 confirms the diagnosis of subclinical spongiform encephalopathy in this animal (*15*) and is the first to demonstrate transmission from a subclinical natural case of spongiform encephalopathy in a bovine species. Also, this report is the first of infectivity in the ileum from a field case of spongiform encephalopathy other than scrapie in sheep.

Apparently low titers or only traces of infectivity were detected in spleen, lung, submandibular salivary gland, conjunctiva, and skin. In bioassays of TSE infectivity, the possibility that trace levels of infectivity in tissues may represent postmortem or laboratory contamination of uninfected tissues with infected material has to be considered. Such an explanation is unlikely in the present study for the following reasons. Many of the tissues which contained only traces of infectivity were taken from kudu A1212, an animal from which tissues were collected by using rigorous sterile procedures to prevent cross-contamination, and no pattern between the order of tissue sampling and the bioassay results from this animal suggests a sequence of tissue contamination. A low incidence of disease in the mice or failure to detect infectivity from tissues previously fixed or processed to paraffin wax may be attributable to a reduced titer of infectivity, which can occur as a result of such treatments. The wide range of survival periods for positive mice in these assays (Table 2) is similar to those seen when brain tissue from confirmed cases of BSE in domestic cattle was injected into C57Bl-J6 mice of the same source as used in the current study (G.A.H. Wells and M. Dawson, unpub. data). These results contrast with those from a previous study (*13*) in which 20 of 21 C57Bl mice were positive for spongiform encephalopathy after injection of formalin-fixed brain from the index case of TSE in greater kudu (kudu A664), with a mean incubation period of 465±14 days (M. Bruce, pers. comm.). We conclude that the low incidence of positive mice in certain tissue groups is due to a lower titer of infectious agent within these tissues when compared with the CNS or ileum.

The distribution of BSE infectivity in tissues of greater kudu contrasts with that in tissues of BSE-infected cattle (*24**,**25*) but is more like the distribution found in genetically susceptible sheep infected with scrapie or experimental BSE (*26**–**28*). In field cases of cattle with BSE, infectivity has been found only in the CNS (*25*), but in cattle experimentally challenged orally with the agent of BSE, ileum and bone marrow have also been shown to contain infectivity (*21**,**24**,**29*). In classical studies of scrapie in sheep and goats, infectivity was detected in the nervous and lymphoreticular systems, placenta, adrenal gland, nasal mucosa, lung, pancreas, liver, bone marrow, thymus, and alimentary tract, although most of the non-neural, non-lymphoreticular peripheral tissues contained only low titers of agent (*30**,**31*).

The finding of infectivity in kudu skin, conjunctiva, and submandibular salivary gland was unexpected as these tissues have not been previously shown to be infective in scrapie, BSE, or any naturally occurring TSE. Nonetheless, infectivity has been found previously in salivary glands of mice after injection of infected tissues with a high titer of scrapie agent (*32*) and of mink injected with the transmissible mink encephalopathy agent (*33*). In experimentally-induced transmissible mink encephalopathy, low concentrations of agent occurred in liver, kidney, intestine, and salivary gland only after replication in the CNS and in some lymphoreticular tissues (*33*). The inconsistent observation of low levels or traces of infection in certain non-neural and non-lymphoreticular tissues is in general a feature of both natural and experimental TSE. Given the relative paucity of data on the tissue distribution of infectivity in TSEs, the finding of infection in any given tissue should probably not be regarded as surprising. The infectivity in certain tissues subsequent to CNS involvement may be a rare event incidental to the pathogenesis of the disease.

We have previously indicated that the epidemiology of BSE in the small kudu herd at London Zoo was consistent with either a particularly high susceptibility to infection, the occurrence of direct animal-to-animal transmission of the disease, or with a combination of these factors (*2**,**14**,**15*). The presence of infectivity in tissues, such as the skin and salivary gland, suggests possible routes by which direct transmission could occur. Eklund et al. (*32*), for example, suggested infection of the salivary gland as an explanation for contact infection of scrapie between mice.

Given the extended survival period range with BSE in the C57Bl-J6 mice used in the current study compared to the incubation periods in C57Bl mice used previously (*13*) and the relative insensitivity of the mouse model (*24*), these results may be an underestimate of the extent of infectivity in the kudu tissues assayed. A recently reported rapid immunoassay shown to be capable of detecting PrP^BSE^ in the brainstems of cattle with a sensitivity similar to that of the infectivity levels determined by end-point titration in Tg(BoPrP) mice (*34*) possibly offers prospects for more sensitive detection of disease-related PrP as a proxy for infectivity bioassay. An important area for further research, therefore, is to investigate whether our results represent true qualitative differences in the biology of BSE in the greater kudu and the domestic cow or possibly indicate similarities, unapparent only because of the variables inherent in the sensitivities of current bioassay methods.
