# Correction Vol. 10, No. 3

**DOI:** 10.3201/eid1007.C311007

**Published:** 2004-07

**Keywords:** errata, erratum, correction

In the article entitled “Distribution of Bovine Spongiform Encephalopathy in Greater Kudu (*Tragelaphus strepsiceros*)” by Andrew A. Cunningham et al., errors occurred in the title.

The corrected title appears below:

Bovine Spongiform Encephalopathy Infectivity in Greater Kudu (*Tragelaphus strepsiceros*).

The corrected article appears online at http://wwwnc.cdc.gov/eid/article/10/6/03-0615_article.htm.

We regret any confusion this error may have caused.

